# *Klebsiella* brain abscess in an immunocompetent patient: a case report

**DOI:** 10.1186/s13256-020-02633-0

**Published:** 2021-02-04

**Authors:** Clay Wu, Semi Han, Ahmet Baydur, Brett Lindgren

**Affiliations:** 1 Division of Pulmonary, Critical Care and Sleep Medicine, University of Southern California, 2020 Zonal Ave, IRD 723, Los Angeles, CA 90033 USA; 2 Department of Internal Medicine, University of Southern California, Los Angeles, California USA

**Keywords:** Case report, *Klebsiella*, Brain abscess, Neurosurgery

## Abstract

**Background:**

*Klebsiella pneumoniae* brain abscesses are a rare entity and typically present in immunocompromised patients. We present a case of an overall healthy patient who developed a *Klebsiella pneumoniae* brain abscess in the absence of liver pathology.

**Case presentation:**

A 46-year-old Vietnamese man with past medical history significant for hypertension presented to the hospital with acute on chronic worsening of altered mental status, personality changes, and gait dysfunction. Initial vitals revealed temperature of 37.1 °C, heart rate 87 beats/minute, blood pressure 150/87 mmHg, respiratory rate 18/minute, and oxygen saturation 99% on room air. Physical exam was notable for altered mental status, Glasgow Coma Scale (GCS) score of 14, and right lower facial droop. Cardiopulmonary exam was within normal limits. Head computed tomography (CT) showed a left frontotemporal mass, with subsequent brain magnetic resonance imaging (MRI) revealing a ring-enhancing lesion concerning for a brain abscess. The abscess was urgently drained; however, there was intraoperative spillage into the ventricles. Intraoperative cultures grew *Klebsiella pneumoniae*, and the patient was maintained on appropriate antibiotics. He developed worsening mental status, septic shock, and cerebral edema requiring decompressive left hemicraniectomy. Computed tomography of the abdomen and pelvis revealed no hepatic lesions. The patient did not improve, and the family elected for comfort measures.

**Conclusion:**

High mortality is associated with *Klebsiella pneumoniae* (as opposed to *Klebsiella oxytoca*) brain abscesses, especially in the setting of intraventricular spread. This case illustrates the need for early detection, and an aggressive medical and surgical treatment approach is required for a potential favorable outcome.

## Introduction

Brain abscesses are life-threatening infections that remain a clinical challenge despite the advancements in imaging, diagnostic techniques, and antibiotic options. In Western countries, community-acquired brain abscesses are relatively rare, with an estimated incidence of 1% to 2%. The majority of pathogens are bacterial, primarily involving *Streptococcus* and *Staphylococcus* spp., while *Klebsiella pneumoniae* (*K. pneumoniae*) has been implicated in less than 1% of cases [1]. *Klebsiella* is a gram-negative bacillus known for causing opportunistic infections. Risk factors for the development of *K. pneumoniae* brain abscess include diabetes mellitus, alcoholism, cirrhosis, and head trauma with craniotomy [2].

We present a case of an immunocompetent Vietnamese American patient who developed a *K. pneumoniae* brain abscess in the absence of predisposing factors. Despite the prompt evacuation and isolation of the pathogen, the patient did not survive the overwhelming sepsis that was further complicated by spillage of abscess material during the procedure.

## Case presentation

The patient was a previously healthy 46-year-old Vietnamese American man who was admitted for progressively worsening altered mental status, incoherent speech, personality changes, and gait dysfunction prior to the COVID-19 pandemic. According to his son, the patient showed mild left facial twitching and bizarre behavior such as locking himself in his bedroom for 4 days prior to admission. The family reported that the patient was not taking any prescription or illicit medications. He held an office job.

On initial presentation, vital signs were temperature 37.1 °C, heart rate 87 beats/minute, blood pressure 150/87 mmHg, respiratory rate 18/minute, and pulse oxygen saturation 99% on room air. His neurologic exam was notable for disorientation, obstinate affect, repeating and mimicking words of the interviewer, and a Glasgow Coma Scale (GCS) of 14. He appeared lethargic and oriented only to self. The patient had right conjugate gaze, right lower facial weakness, and right dysmetria. Otherwise cardiopulmonary examination was within normal limits.

**Fig. 1:**
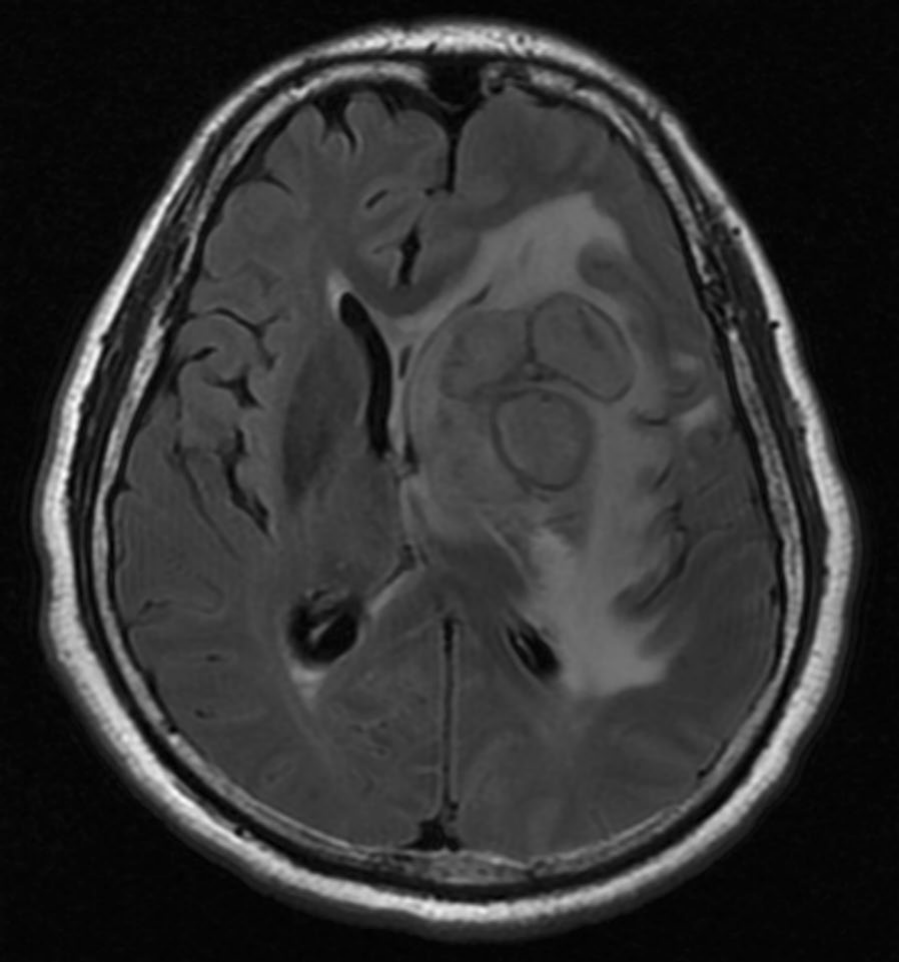
Magnetic resonance imaging of the brain showing a T1 hyperintense, T2 hyperintense heterogeneous lesion with loss of signal on fat-saturated T1 sequence involving right Meckel cave and posterior cerebellopontine angle measuring approximately 3.3 × 1.8 × 1.4 cm

Initial workup ruled out metabolic and toxicologic etiologies. Serologic testing for human immunodeficiency virus, hepatitis, diabetes, cirrhosis, and immunoglobulins were negative. Brain magnetic resonance imaging (MRI) revealed a large mass (4.2 cm × 4.0 cm × 3.9 cm) involving the left frontal and temporal lobes with vasogenic edema, 1.1 cm midline shift, and mild hydrocephalus (Fig. 1). This ring-enhancing left frontotemporal mass was concerning for brain abscess. He received broad-spectrum antibiotics, dexamethasone for vasogenic edema, and underwent immediate neurosurgical drainage of the large abscess within 4 hours of presentation. Unfortunately, there was intraoperative spillage of the pus into the ventricles, and the patient developed significant swelling of the surrounding brain from cerebritis. Despite appropriate antibiotic coverage and supportive care in the intensive care unit, his abscess persisted, requiring external ventricle drain placement and decompressive left hemicraniectomy. The susceptibility from the blood cultures and cerebrospinal fluid cultures indicated intravenous ceftriaxone to treat *K. pneumoniae*. Computed tomography (CT) of the abdomen and pelvis revealed no hepatic lesions or biliary ductal dilatation. The patient's clinical status continued to decline due to refractory intracranial hypertension and multi-organ failure secondary to sepsis. Ultimately, the family elected to pursue comfort care, and the patient died on day 14 of hospitalization.

## Discussion

*Klebsiella* is a rare cause of community-acquired brain abscess and is associated with a mortality rate up to 27%, which increases to 38% with complicating intraventricular empyema. We present a case of *Klebsiella* brain abscess in an immunocompetent Asian American man without prior hepatic abscess or other predisposing factors.

This patient’s presenting GCS of 14 and lack of immunodeficiency and clinically significant comorbidities are good prognostic factors in the setting of a brain abscess. Some studies have shown that a presenting GCS greater than 12 is associated with a significantly better outcome [3, 4]. However, our patient presented with a deep-seated periventricular abscess and preoperative hydrocephalus, which portend a poor outcome. Brain abscesses located within 7 mm of the ventricles are more likely to have intraventricular rupture leading to fatal complications, with mortality rates of 38.7–80% [5]. The risk of rupture into the ventricle or subarachnoid leakage of pus during surgical intervention leading to ventriculitis or meningitis, as seen in this patient, has been reported in the literature, though the prevalence during aspiration or excision is not well documented.

Surgical intervention over medical management was appropriate for the patient given the large size of the abscess [3]. In addition to antibiotics, the patient was given dexamethasone for vasogenic edema. There is no clear guideline on the use of steroids in treating bacterial brain abscesses. One meta-analysis showed that the addition of dexamethasone to treatment for patients with brain abscesses was not associated with increased mortality [4]. The indication of dexamethasone administration in cases of brain abscess remains at the treating physician’s discretion.

This case was complicated by postoperative *Klebsiella* cerebritis and ventriculitis, which dramatically increases the incidence of death. His case was treated solely with intravenous antibiotics. Multiple studies have demonstrated that intraventricular (IVT) and intrathecal (IT) antibiotics can shorten the period to cerebrospinal fluid sterilization with gram-negative bacterial infection of the central nervous system in the setting of treatment-refractory post-neurosurgical meningitis/ventriculitis [5, 6]. However, an IVT or IT approach should be used with caution given the risk of chemical meningitis and seizures [7]. It is unclear whether empirical treatment with IVT or IT antibiotics at the time of drain placement is beneficial, but it could have been considered in this case.

## Conclusion

While *K. pneumoniae* has previously been described as an important pathogen in immunocompromised patients who develop brain abscess, this case demonstrates that this pathogen must also be considered in immunocompetent patients. Even with timely neurosurgical intervention and antibiotics, patients may have an unfavorable outcome.

## Data Availability

Not applicable.

## Abbreviations

GCS: Glasgow Coma Scale
CT: Computed tomography
MRI: Magnetic resonance imaging
IVT: Intraventricular
IT: Intrathecal
